# Risk Factors, Incidence, and Management of Re-Injury following Repair of Shoulder Rotator Cuff

**DOI:** 10.26502/josm.511500193

**Published:** 2025-03-31

**Authors:** David Parvizi, Ramtin Sahafi, Timothy Pisarski, Sugeeth Kandikattu, Manas Aavula, Devendra K Agrawal

**Affiliations:** Departments of Translational Research, College of Osteopathic Medicine of the Pacific, Western University of Health Sciences, Pomona, California 91766 USA

**Keywords:** Biological scaffolds, Diabetes, Fatty infiltration, Hyperlipidemia, Muscle atrophy, Risk factors, Rotator cuff re-injury, Rotator cuff tear, Surgical repair, Tendon healing

## Abstract

Rotator cuff tears are among the most common musculoskeletal injuries worldwide, often requiring surgical intervention to restore shoulder function. Despite improvements in surgical techniques, rotator cuff re-injury remains a significant challenge, influenced by a combination of patient-related and procedural factors. The incidence of re-injury after surgery ranges from 15% to 21%, varying based on the severity of the initial injury and adherence to rehabilitation. In this article, we critically examine the risk factors, incidence, and management strategies associated with rotator cuff re-injury. Key risk factors include advanced age, larger tear size, poor tissue quality, high activity levels, and comorbid conditions like diabetes and hyperlipidemia. Age-related degenerative changes, muscle atrophy, and fatty infiltration impair tendon healing, increasing the risk of re-injury. Emerging geometric classifications of rotator cuff tears (Types 1–4) provide valuable insights into prognosis and guide surgical approaches. Management strategies for re-injury include both conservative approaches, such as physical therapy and activity modification, and surgical revisions, including tendon transfers and superior capsular reconstruction. Novel interventions like biological scaffolds, mesenchymal stem cell therapy, and machine learning-driven rehabilitation protocols are being explored to enhance tendon healing and reduce re-injury rates. However, gaps remain in understanding the biological mechanisms of tendon repair and optimizing personalized treatment strategies. Future research should focus on integrating biomolecular insights with clinical practice to improve outcomes and reduce the burden of rotator cuff re-injury.

## 1. Introduction

Rotator cuff tears are one of the most prevalent types of musculoskeletal injuries that require surgical intervention with more than two million Americans each year suffering from some type of rotator cuff injury [1]. Despite the significant advances made in treatment of rotator cuff tears, risk of re injury continues to be a challenging concern. Muscles involved in the rotator cuff are the infraspinatus, teres minor, subscapularis, and supraspinatus. Each of these muscles works together to stabilize motions such as glenohumeral abduction, internal rotation, and external rotation. Injuries to the rotator cuff generally occur due to trauma, repeated use, or degenerative changes which can occur over time [2]. Furthermore, the supraspinatus muscle has been documented as the most likely to become susceptible to injury [3].

Symptoms of rotator cuff tears include pain in the shoulder while initiating movement, weakness in the affected arm, and limited range of motion especially in external and internal rotation. Standard of care treatment for rotator cuff re-injury includes physical therapy or arthroscopic rotator cuff repair followed by post-operative rehabilitation [4].

Age, tear size, tissue quality, activity level, compliance with rehabilitation, and smoking have been found to be the biggest factors contributing to re-injury [5]. Of these factors, age is the biggest predictor of rotator cuff retear due to degenerative tissue quality changes and reduced healing capacity [6]. Moreover, as one ages the probability of developing comorbidities increases significantly. Diabetes, hyperlipidemia, smoking, and osteoporosis make up the most significant factors as predictors for age-related rotator cuff retear [7]. Over time, muscle atrophy and fatty infiltration due to chronic rotator cuff tear can also play a role in the probability of rotator cuff retear. The presence of atrophy and fatty infiltration further compromises tissue quality and reduces the ability for tendon healing significantly [8–10]. Additionally, immune response due to several inflammatory mediators [9–12] and the regulation and phenotype of collagens are critical in the healing response of tendon following initial injury and repair [13,14] and could result in significant pain and re-tear [15,16].

There is no standard classification for rotator cuff tear size. However, there are new geometric classifications consisting of Type 1, Type 2, Type 3, and Type 4. Type 1 rotator cuff tears are classified as having a predictable and good prognosis. Type 2 is described as a longitudinal-shaped tear and is repaired side to side with good prognosis. Type 3 tears are considered massive tears in which coronal and sagittal dimensions measure greater than 2 × 3 cm. These types of tears are generally treated with interval slides or partial repair, and prognosis can vary from person to person. Lastly, Type 4 tears are characteristic of end-stage degenerative tears of the glenohumeral joint in which there is articulation between the humeral head and the acromion process, making the rotator cuff tear irreparable. Type 4 classification tears include complete tears of superior and posterior rotator cuff, supraspinatus, infraspinatus, and teres minor [17].

Activity level has been shown to have a large impact on retear rates for rotator cuff injury in patients presenting before or after surgical repair. High activity individuals such as athletes and manual laborers are at the highest risk for rotator cuff retear. Sports such as baseball, tennis, swimming, and jobs that involve heavy lifting and/or repeated overheard shoulder use have a significant risk in retear. Following surgery, those who participate in physically demanding jobs and high impact sports have higher rates of rotator cuff retear after surgery [18]. Of the occupations that require repetitive shoulder use, carpenters, electricians, and construction workers were found to have higher rotator cuff retear post-surgery compared to those returning to jobs that do not require physical work [19]. For high activity individuals, it is recommended to undergo a long-term, structured, rehabilitation program under the supervision of a physician and physical therapist with a gradual increase in activity over time to decrease the risk of rotator cuff retear [20].

The primary objective of this study is to evaluate an array of risk factors that contribute to occurrence of rotator cuff re-injury, assess existing gaps in knowledge, and comprehensively explore treatment modalities in addressing instances of rotator cuff re-injury.

## 2. Incidence

Rotator cuff injuries can differ significantly based on factors such as age, level of activity, and fitness status. In the United States, the prevalence of rotator cuff tears ranges from 7% to 22% for adults over the age of 40 [21]. Moreover, rotator cuff re-injury is one of the most common complications of rotator cuff injuries. The incidence of rotator cuff re-injury after surgery ranges from 15% to 21% between 6 months to 24 months after surgery. Specifically, the re-injury rates differ based on the time frame after surgery. Within 3 months after surgery, retear incidence is about 15%. Between 3 to 6 months retear rates are at 21%. From 6 to 12 months retear rates are at 16%. These variations in retear rates are attributable to original size of the rotator cuff tear, age, and surgical technique used [22].

Rotator cuff tears are most prone to re-injury within the first six months of the initial injury. Several factors can hinder tendon healing, including age, tear size, tendon retraction, and fatty infiltration [23]. The literature on rotator cuff retear remains insufficient on a broad scale. However, numerous smaller studies have examined retear incidences within hospital and outpatient settings. A study conducted by Smid, Hart, and Puskeilier [24] to assess the predictability of rotator cuff re-injury included 37 shoulders with rotator cuff tears. 21 of the 37 shoulders included a tangent sign, which is defined as a failure of the supraspinatus to cross a line from the superior border of the coracoid process to the superior border of the scapular spine. Before surgery, 18 of the 21 shoulders with tangent signs were shown to have a recurrence of rotator cuff tear within 2 years. The average tear size for shoulders with a positive tangent sign was 39.6 mm and the average retear size for the subjects was 40.8 mm [24]. Another study which examined rotator cuff retear rate looked at 693 patients who underwent arthroscopic rotator cuff repair. The study showed that the rate of retear was 7.22% of those 693 patients. Moreover, this study found that rotator cuff retears were most affected by age, presence of inflammatory arthritis, completeness of the rotator cuff repair, the initial tear size, and mean operative time [25].

## 3. Risk Factors

Following a rotator cuff tear repair, there are four major factors that increase the risk of developing a recurrent rotator cuff injury: age, tear size, diabetes and hyperlipidemia.

### Age:

Age is a well-documented risk factor for rotator cuff retears. In a meta-analysis of 31 studies completed by Longo, U.G., et al, a group of patients ranging from 53 to 67 years old was statistically analyzed for the risk of retear following a rotator cuff repair surgery [26]. After splitting this cohort into two groups ranging from 53 to 60 years old and 61 to 67 years old, the risk of retear for the younger cohort was 14.4% and 24.3%, respectively, yielding a statistically significant difference (P<0.0001), indicating a correlation between older age and rotator cuff retear. Another meta-analysis including 38 studies with a total of 3072 patients found that the risk of rotator cuff retear doubled from 15% at 50 years old to 31% at 70 years old [27]. These poorer outcomes following a rotator cuff repair in older adults suggests a negative association between the structural integrity of the rotator cuff after repair and age of the patient, likely contributing to the greater prevalence of retears in older adults. However, in a study by Routledge, et. al, age was not found to be the largest risk factor for rotator cuff re-injury, contrary to many studies, suggesting multifactorial causation for rotator cuff re-injury [28].

### Tear Size:

Tear size is a significant predictor of rotator cuff re-injury after surgical repair. Larger tears are generally associated with higher failure rates, likely due to the increased biomechanical stress required for healing across a broader area and the greater degenerative changes often observed in these cases [29, 30]. Additionally, studies have shown that tear size impacts the integrity of postoperative repair, with larger tears more likely to experience incomplete healing, leading to an increased likelihood of re-injury [31]. The increased difficulty in achieving a secure fixation and the higher stress required during rehabilitation for larger tears underscore the importance of early intervention and targeted rehabilitation protocols for patients with large rotator cuff injuries. In terms of tear size, there are three classifications: small (less than 1 cm), medium (1–3 cm), large (3–5 cm), and massive (greater than 5 cm). Small to medium sized rotator cuff tears are less prone to retear and thus smaller tears have better prognosis compared to large and massive tears in which retear rates are significantly higher [32]. For large and massive tears, retear rates can vary between 50–94%, especially if there is poor tissue quality [33].

### Diabetes:

As a result of diabetes, there is a higher risk of rotator cuff retear, impaired tendon healing, reduced functional recovery, and increased intraoperative complications. Moreover, diabetes patients are twice as likely to experience complications following arthroscopic rotator cuff repair, which includes double the risk of retear and repair failure [34]. In a study that compared diabetic-induced rats to non-diabetic rats, evaluation of the Achilles tendon after an Achilles tendon repair had shown that in the diabetic rats, there was a lower peak force for failure after 2, 4, and 6 weeks follow up. Histologically, the diabetes-induced rats had significantly less fibroblast proliferation and lymphocyte infiltration compared to the control [35].

As mentioned, diabetes increases the risk of rotator cuff retear by negatively impacting the body’s ability to heal itself. Chronic hyperglycemia leads to an increase in the amount of glycation end products (AGEs) which adversely affects collagen quality, further impacting the structural integrity of the tendon [13,15]. Moreover, diabetes induces chronic inflammation and vascular dysfunction resulting in less blood supply and nutrients being delivered to the already-damaged tendon. Studies have shown that diabetic patients have higher rates of postoperative retears. One meta-analysis reported retear rates of 24.5% in diabetic patients compared to 13.7% in non-diabetic patients [36].

### Hyperlipidemia:

Additionally, hyperlipidemia impacts tendon to bone healing by allowing for increased fatty infiltration of the rotator cuff muscles leading to increased retear rates. As discussed by Sripathi et al. [37], metabolic syndrome predisposes tendon repairs to retears due to increased inflammation, impaired collagen reorganization, and tendon stiffness secondary to fatty infiltration. Similarly, Fang et al. [38] observed disorganized collagen fibers in hyperlipidemic swine, reinforcing the effect that metabolic disorders have on the integrity and stability of tendons. In a similar manner, numerous studies have observed reduced elasticity and viscoelastic properties of repaired rotator cuff tendons in hyperlipidemic swine [39,40,41]. This finding further emphasizes the significant role of metabolic diseases in influencing the outcomes of tendon tear repair. Metabolic syndrome related risk factors for retear prevention includes glucose and cholesterol management, which has been shown to blunt the risk of retear [42,43].

## 4. Management of Re-injury in the Rotator Cuff

Re-injury proceeding a rotator cuff repair portrays a wide range of recurrence, ranging from 13% to 94% [44]. As a result, effective management of re-injury requires a multifactorial approach that is patient-centered and includes assessing functional requirements, causes of the initial failure, and the possibilities of treatment. Several factors increase the risk of retear including age, shoulder anatomy, and medical comorbidities such as diabetes, hyperlipidemia, and osteoporosis. For instance, patients older than 80 years have a very high retear rate (seven-fold) as compared to younger patients [45]. Management of these risk factors is pivotal for the initial care as well as prophylaxis for re-injury.

Rotator cuff re-injury management is characterized by two categories: conservative or surgical. Patients with insignificant symptoms or those who cannot undergo surgery may benefit from a conservative approach, which involves physical therapy aimed at reducing rotator cuff stress to enhance their functionality. The primary objective is to restore the rotator cuff’s function while simultaneously minimizing any potential structural damage. If the re-injury is irreparable, some patients might qualify for superior capsular reconstruction or tendon transfer [46]. If a patient’s tendon integrity is sufficient, revision rotator cuff repair is a viable option.

**Figure 1:**
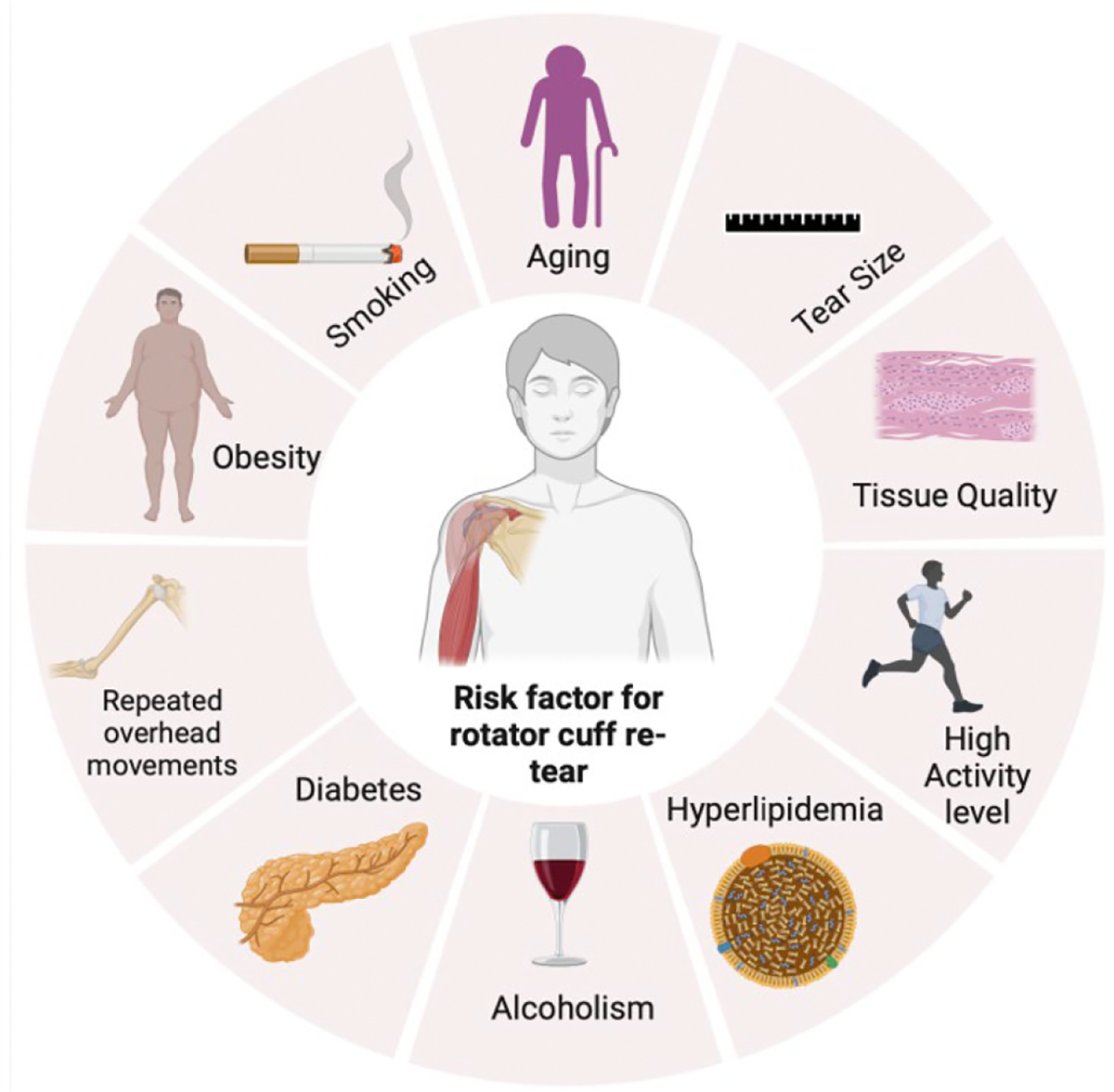
The risk of rotator cuff re-injury is influenced by multiple factors, including tear size, with larger tears having a higher likelihood of recurrence due to compromised tendon integrity. Systemic conditions like diabetes, hyperlipidemia, alcoholism, and obesity can impair healing by reducing blood flow and collagen synthesis, while aging further weakens tendon structure and regenerative capacity. Additionally, a high activity level may increase mechanical stress on the repaired tendon, further elevating the risk of re-tear.

Rehabilitation plays an important role both in dealing with the initial surgical repair and avoiding re-injury. Along with surgical procedures, immobilization is helpful to ensure proper healing. Gradual progression to passive range-of-motion exercises improves flexibility without overstressing the repair site, while active range-of-motion strengthening exercises are introduced later a patient-to-patient basis. Rehabilitation protocols should be tailored to everyone’s needs, as engaging in improper or premature activities can increase the risk of sustaining additional injuries [47]. Treating modifiable risk factors such as smoking cessation and optimizing metabolic health, in conjunction with physical therapy, can also improve outcomes. Ultimately, a successfully managed treatment plan that results in a return to normalcy after a rotator cuff repair relies on an intersected approach that incorporates surgical expertise, rehabilitation, and patient education to enhance long-term outcomes (Figure 1).

## 5. Gaps in Knowledge

Although rehabilitation strategies for rotator cuff tears have advanced significantly, there are still numerous persisting gaps that underscore the limitations of the current understanding of treatment techniques and surgical methods. A pivotal limitation in management of rotator cuff tears entails the process of tendon healing after surgical repair. Tendon healing is a multifactorial process in which multiple intersecting biological responses must be considered. While inflammatory responses and intracellular pathways are characterized as critical processes, the inter-relatedness between inflammation and collagen remodeling is poorly understood which can significantly limit potential restorative interventions [48]. Additionally, comorbidities and risk factors like osteoarthritis, diabetes, hyperlipidemia, and smoking can complicate the objective assessment of tendon healing and collagen remodeling due to their additive nature. Diabetic patients portray altered collagen characteristics as well as diminished vascular properties which increases the risk of compromised tendon integrity [49]. The use of biological stem cells, scaffolds, and grafts in rotator cuff tears holds promise for enhancing tissue regeneration. However, the full extent of their long-term potency remains undiscovered, and the unexplored cost-effectiveness of these treatments poses a significant obstacle to standardizing regenerative medicine for rotator cuff tears [50]. Limitations in classifying rotator cuff tears based on lifestyle differences among patient populations hinder the development of an optimal treatment approach for individuals with high levels of rotator cuff activity and intensity, such as athletes and laborers [51]. To address these barriers, a cross-functional approach should be administered that intersects biomolecular, biomechanical, and patient-oriented approaches to maximize the potency and cost-effectiveness of new and emerging treatment methods for rotator cuff tears. To determine the effectiveness and safety of new treatments and to improve rehabilitation methods, large, well-conducted, randomized controlled trials are required. Bridging the gaps in assessing tendon quality and the healing process may also provide a better understanding of the higher recurrence rates of certain tendon tears. This knowledge can help identify at-risk populations and refine strategies to reduce the incidence of retears in those groups.

## 6. Future Directions

Rotator cuff retear has several treatment modalities including surgical repair and non-surgical management through physical therapy, rehabilitation, pain management, and activity/lifestyle modifications. Pain management generally involves the use of NSAIDs or corticosteroid injections. Moreover, mesenchymal stem cell therapy holds promise as a potential alternative for managing rotator cuff tears. However, studies have found over and over that the use of mesenchymal stem cells have had very few clinical successes. Furthermore, there are substantial obstacles to its widespread adoption, including the absence of cost standardization and a lack of affordability [52].

Scaffolds and grafts have emerged as a more promising therapy, demonstrating a reduction in the rate of rotator cuff retear. Biological or synthetically made scaffolds can be used to reinforce tendon damage and aid in tissue regeneration. One study found that synthetic scaffolds, like Ligament Advanced Reinforcement System (LARS), which is an artificial ligament used as a synthetic graft for reconstruction, showed exceptionally promising results after 36 months of follow-up. All patients reported a significant reduction in pain, improved rotator cuff function, and increased angular range of motion with intact tendons in 15 out of 17 patients [53]. Innovative methods, such as 3D-printed scaffolds and bioengineered grafts, hold the potential to revolutionize tendon repairs. Recent studies have shown that 3D-printed scaffolds can be meticulously engineered to replicate the natural tendon structure by incorporating chemical features that enhance cell adhesion and proliferation [54]. These technologies, in conjunction with the use of growth factors, could greatly improve tendon regeneration and decrease the rate of retear. Furthermore, the integration of machine learning and artificial intelligence in clinical practice has paved the way for the implementation of personalized treatment plans tailored to individual patient characteristics. For instance, algorithms trained on diverse patient datasets through machine learning can predict the risk of retear and generate customized rehabilitation protocols [55]. These innovative approaches, coupled with ongoing analysis of tendon biology and biomechanical integrity, have the potential to significantly enhance patient outcomes and alleviate the financial burden associated with rotator cuff injuries. By addressing these concerns, healthcare systems can develop more effective strategies for managing rotator cuff tears, thereby reducing the severity and likelihood of re-injury among patients.

## 7. Conclusion

Although shoulder rotator cuff injury repairs are a common surgical procedure, the risk of retear remains a significant complication. This paper examines several key risk factors associated with retear of rotator cuff injuries, including patient age, tear size, diabetes, hyperlipidemia, surgical technique, tendon size, and rehabilitation protocols. By comprehending how these factors interact, physicians can develop personalized treatment plans to minimize the risk of rotator cuff retears. The incidence of retear varies among patients. However, advancements in surgical techniques and improved post-operative care have contributed to reducing the risk of rotator cuff retear.

## 8. Key points

- Rotator cuff tears can severely restrict shoulder mobility and interfere with daily activities, often requiring medical intervention for recovery. Despite advancements in surgical techniques, many patients face a risk of re-injury within two years of the initial repair.
- Factors such as older age, larger tear dimensions, compromised tissue quality, high physical demands, and metabolic conditions like diabetes and hyperlipidemia increase the risk of re-injury.
- Deterioration in muscle structure, including atrophy and fatty deposits, reduces the ability of tendon to heal effectively after surgery.
- Classifying rotator cuff tears based on geometric patterns helps clinicians predict outcomes and choose the most appropriate surgical method.
- Non-surgical treatments, including customized physical therapy plans and lifestyle adjustments, play a vital role in managing rotator cuff injuries and preventing re-injury.
- For recurrent injuries, surgical options like tendon transfers, superior capsular reconstruction, or repeat rotator cuff repairs may be considered depending on the patient’s condition.
- Cutting-edge therapies, such as tissue scaffolds, stem cell applications, and artificial intelligence-driven rehab programs, are being explored to improve healing processes and reduce the likelihood of future tears.

## References

[1] Lewis J Rotator cuff related shoulder pain: Assessment, management and uncertainties. Man Ther 23 (2016): 57–68. 27083390 10.1016/j.math.2016.03.009

[2] Tashjian RZ. Epidemiology, natural history, and indications for treatment of rotator cuff tears. Clin Sports Med 31 (2012): 589–604. 23040548 10.1016/j.csm.2012.07.001

[3] Bandara U, An VVG, Imani S, Rehabilitation protocols following rotator cuff repair: a meta-analysis of current evidence. ANZ J Surg 91 (2021): 2773–2779. 34582083 10.1111/ans.17213

[4] Matlak S, Andrews A Looney A, Postoperative Rehabilitation of Rotator Cuff Repair: A Systematic Review. Sports Medicine and Arthroscopy Review 29 (2021): 119–129. 33972488 10.1097/JSA.0000000000000310

[5] Swansen T, Wright MA, Murthi AM. Postoperative Rehabilitation Following Rotator Cuff Repair. Phys Med Rehabil Clin N Am 34 (2023): 357–364. 37003657 10.1016/j.pmr.2022.12.003

[6] Le BT, Wu X, Lam PH, Factors predicting rotator cuff retears: an analysis of 1000 consecutive rotator cuff repairs. The American Journal of Sports Medicine 42 (2014): 1134–1142. 24748610 10.1177/0363546514525336

[7] Tashjian RZ. Epidemiology, natural history, and indications for treatment of rotator cuff tears. Clin Sports Med 31 (2012): 589–604. 23040548 10.1016/j.csm.2012.07.001

[8] Gladstone JN, Bishop JY, Lo IK, Fatty infiltration and atrophy of the rotator cuff do not improve after rotator cuff repair and correlate with poor functional outcome. Am J Sports Med 35 (2007): 719–28. 17337727 10.1177/0363546506297539

[9] Le H, Rai V, Agrawal DK. Inflammation and Fatty Infiltration Correlates with Rotator Cuff Muscle Atrophy in Hypercholesterolemic Yucatan Microswine. J Orthop Sports Med 6 (2024): 198–213. 39639857 10.26502/josm.511500161 PMC11619632

[10] Thankam FG, Dilisio MF, Agrawal DK. Immunobiological factors aggravating the fatty infiltration on tendons and muscles in rotator cuff lesions. Mol Cell Biochem 417 (2016): 17–33. 27160936 10.1007/s11010-016-2710-5

[11] Thankam FG, Roesch ZK, Dilisio MF, HMGB1 priming triggers NLRP3 inflammasomes in the rotator cuff tendon injury. Sci. Reports (Nature) 8 (2018): 8918. 10.1038/s41598-018-27250-2 PMC5995925 29891998

[12] Connor D, Thankam FG, Agrawal DK. Karyopherins in the remodeling of extracellular matrix: Implications in tendon injury. J Ortho Sports Med 5 (2023): 357–374. 10.26502/josm.511500122 PMC10569131 37829147

[13] Rajalekshmi R, Agrawal DK. Understanding Fibrous Tissue in the Effective Healing of Rotator Cuff Injury. J Surg Res (Houst) 7 (2024): 215–228. 38872898 10.26502/jsr.10020363 PMC11174978

[14] Singh D, Rai V, Agrawal DK. Regulation of collagen I and collagen III in tissue injury and regeneration. Cardiol Cardiovasc Med 7 (2023): 5–16. 36776717 10.26502/fccm.92920302 PMC9912297

[15] Rai V, Deepu V, Agrawal DK. Targeting RAGE-signaling pathways in the repair of rotator-cuff injury. Mol Cell Biochem (2024). 10.1007/s11010-024-05132-8 PMC11961478 39395136

[16] Raney EB, Thankam FG, Dilisio MF, Pain and the pathogenesis of biceps tendinopathy. Am J Translational Res 9 (2017): 2668–2683. PMC5489872 28670360

[17] Namdari S, Henn RF 3rd, Green A. Traumatic anterosuperior rotator cuff tears: the outcome of open surgical repair. J Bone Joint Surg Am 90 (2008): 1906–13. 18762651 10.2106/JBJS.F.01446

[18] Longo UG, Carnevale A, Piergentili I, Retear rates after rotator cuff surgery: a systematic review and meta-analysis. BMC Musculoskelet Disord 22 (2021): 749. 34465332 10.1186/s12891-021-04634-6 PMC8408924

[19] Kuhn JE, Dunn WR, Sanders R, Effect of rotator cuff re-tear on outcome: A systematic review and meta-analysis. Journal of Bone and Joint Surgery 95 (2013): 591–600.

[20] Denard PJ, Jiwani AZ, Lädermann A, Long-term outcome of arthroscopic massive rotator cuff repair: the importance of double-row fixation. Arthroscopy 28 (2012): 909–15. 22365267 10.1016/j.arthro.2011.12.007

[21] Vidal C, Lira MJ, de Marinis R. Increasing incidence of rotator cuff surgery: A nationwide registry study in Chile. BMC Musculoskelet Disord 22 (2021): 1052. 34930197 10.1186/s12891-021-04938-7 PMC8690465

[22] Lapner P, Henry P, Athwal GS, Treatment of rotator cuff tears: a systematic review and meta-analysis. J Shoulder Elbow Surg 31 (2022): e120–e129. 34906681 10.1016/j.jse.2021.11.002

[23] Rossi LA, Chahla J, Verma NN, Rotator Cuff Retears. JBJS Rev 8 (2020): e0039. 31899699 10.2106/JBJS.RVW.19.00039

[24] Smíd P, Hart R, Puskeiler M. Tangent sign - spolehlivý prediktor rizika reruptury při rekonstrukci šlach svalů rotátorové manžety [Tangent sign - a reliable predictor of risk for tendon re-rupture in rotator cuff repair]. Acta Chir Orthop Traumatol Cech 81 (2014): 227–32. 24945392

[25] Lee YS, Jeong JY, Park C-D, Evaluation of the Risk Factors for a Rotator Cuff Retear After Repair Surgery. The American Journal of Sports Medicine 45 (2017): 1755–1761. 28319431 10.1177/0363546517695234

[26] Villatte G, Erivan R, Nourissat G, Allograft and autograft provide similar retear rates for the management of large and massive rotator cuff tears: a review and meta-analysis. Knee Surg Sports Traumatol Arthrosc 30 (2022): 2039–2059. 34586436 10.1007/s00167-021-06745-y

[27] Khazzam M, Sager B, Box HN, The effect of age on risk of retear after rotator cuff repair: a systematic review and meta-analysis. JSES Int 4 (2020): 625–631. 32939497 10.1016/j.jseint.2020.03.014 PMC7479041

[28] Routledge JC, Saber AY, Pennington N, Re-Tear Rates Following Rotator Cuff Repair Surgery. Cureus 15 (2023): e34426. 36874651 10.7759/cureus.34426 PMC9981227

[29] Orozco E, Dhillon J, Keeter C, Rotator Cuff Repair with Patch Augmentation Is Associated With Lower Retear Rates for Large Tears: A Systematic Review of Randomized Controlled Trials. Arthroscopy 40 (2024): 1300–1308. 37734446 10.1016/j.arthro.2023.08.072

[30] Khair MM, Lehman J, Tsouris N, A Systematic Review of Preoperative Fatty Infiltration and Rotator Cuff Outcomes. HSS J 12 (2016): 170–6. 27385947 10.1007/s11420-015-9465-5 PMC4916083

[31] Paolucci T, Agostini F, Conti M, Comparison of Early versus Traditional Rehabilitation Protocol after Rotator Cuff Repair: An Umbrella-Review. J Clin Med 12 (2023): 6743. 37959210 10.3390/jcm12216743 PMC10650668

[32] Nho SJ, Shindle MK, Sherman SL, Systematic review of arthroscopic rotator cuff repair and mini-open rotator cuff repair. J Bone Joint Surg Am 89 (2007): 127–36. 17908878 10.2106/JBJS.G.00583

[33] Mall NA, Lee AS, Chahal J, An evidenced-based examination of the epidemiology and outcomes of traumatic rotator cuff tears. Arthroscopy 29 (2013): 366–76. 23290186 10.1016/j.arthro.2012.06.024

[34] Borton Z, Shivji F, Simeen S, Diabetic patients are almost twice as likely to experience complications from arthroscopic rotator cuff repair. Shoulder Elbow 12 (2020): 109–113. 32313560 10.1177/1758573219831691 PMC7153207

[35] Egemen O, Ozkaya O, Ozturk MB, The biomechanical and histological effects of diabetes on tendon healing: experimental study in rats. J Hand Microsurg 4 (2012): 60–4. 24293952 10.1007/s12593-012-0074-y PMC3509287

[36] Yang L, Zhang J, Ruan D, Clinical and Structural Outcomes After Rotator Cuff Repair in Patients with Diabetes: A Meta-analysis. Orthop J Sports Med 8 (2020): 2325967120948499. 32995347 10.1177/2325967120948499 PMC7502797

[37] Sripathi P, Agrawal DK. Rotator Cuff Injury: Pathogenesis, Biomechanics, and Repair. J Orthop Sports Med 6 (2024): 231–248. 39574962 10.26502/josm.511500167 PMC11580759

[38] Fang W, Sekhon S, Teramoto D, Pathological alterations in the expression status of rotator cuff tendon matrix components in hyperlipidemia. Mol Cell Biochem (2022). 10.1007/s11010-022-04643-6 36576716

[39] Merlin Rajesh Lal LP, Radwan MM, Thankam FG, Rotator Cuff Tendon Repair after Injury in Hyperlipidemic Swine Decreases Biomechanical Properties. J Ortho Sports Med 5 (2023): 398–405. 10.26502/josm.511500127 PMC10756634 38161622

[40] Merlin Rajesh Lal LP, Agrawal DK. Hyperlipidemia Lowers the Biomechanical Properties of Rotator Cuff Tendon. J Ortho Sports Med 5 (2023): 391–397. 10.26502/josm.511500126 PMC10655854 37982013

[41] Le H, Rai V, Agrawal DK. Inflammation and Fatty Infiltration Correlates with Rotator Cuff Muscle Atrophy in Hypercholesterolemic Yucatan Microswine. J Orthop Sports Med 6 (2024): 198–213. 39639857 10.26502/josm.511500161 PMC11619632

[42] Cho NS, Moon SC, Jeon JW, The influence of diabetes mellitus on clinical and structural outcomes after arthroscopic rotator cuff repair. Am J Sports Med 43 (2015): 991–7. 25622985 10.1177/0363546514565097

[43] Yazdani AN, Rai V, Agrawal DK. Rotator cuff health, pathology, and repair in the perspective of hyperlipidemia. J Orthop Sports Med 4 (2022): 263–275. 36381991 10.26502/josm.511500063 PMC9648405

[44] MacLean IS, Brockmeier SF. Failed and Revision Rotator Cuff Repair. Clin Sports Med 42 (2023): 141–155. 36375866 10.1016/j.csm.2022.08.006

[45] Mandaleson A Re-tears after rotator cuff repair: Current concepts review. J Clin Orthop Trauma 9 (2021): 168–174. 10.1016/j.jcot.2021.05.019 PMC8170498 34123722

[46] Khair MM, Lehman J, Tsouris N, A Systematic Review of Preoperative Fatty Infiltration and Rotator Cuff Outcomes. HSS J 12 (2016): 170–6. 27385947 10.1007/s11420-015-9465-5 PMC4916083

[47] Massachusetts General Hospital. Rehabilitation protocol for rotator cuff repair. MGH Orthopaedics and Sports Medicine Division (2023).

[48] Jiang F, Zhao H, Zhang P, Challenges in tendon-bone healing: emphasizing inflammatory modulation mechanisms and treatment. Front Endocrinol (Lausann) 15 (2024): 1485876. 10.3389/fendo.2024.1485876 PMC11576169 39568806

[49] Bedi A, Fox AJ, Harris PE, Diabetes mellitus impairs tendon-bone healing after rotator cuff repair. J Shoulder Elbow Surg 19 (2010): 978–88. 20303293 10.1016/j.jse.2009.11.045 PMC5257255

[50] Karuppaiah K, Sinha J. Scaffolds in the management of massive rotator cuff tears: current concepts and literature review. EFORT Open Rev 4 (2019): 557–566. 31598334 10.1302/2058-5241.4.180040 PMC6771075

[51] Bandara U, An VVG, Imani S, Rehabilitation protocols following rotator cuff repair: a meta-analysis of current evidence. ANZ J Surg 91 (2021): 2773–2779. 34582083 10.1111/ans.17213

[52] Rynecki ND, Pereira DS. The Role of Mesenchymal Stem Cells in Augmenting Rotator Cuff Repairs. Bull Hosp Jt Dis 76 (2018): 232–237. 31513507

[53] Karuppaiah K, Sinha J. Scaffolds in the management of massive rotator cuff tears: current concepts and literature review. EFORT Open Rev 4 (2019): 557–566. 31598334 10.1302/2058-5241.4.180040 PMC6771075

[54] Jiang X, Wu S, Kuss M, 3D printing of multilayered scaffolds for rotator cuff tendon regeneration. Bioact Mater 5 (2020): 636–643. 32405578 10.1016/j.bioactmat.2020.04.017 PMC7212184

[55] Lalehzarian SP, Gowd AK, Liu JN. Machine learning in orthopaedic surgery. World J Orthop 12 (2021): 685–699. 34631452 10.5312/wjo.v12.i9.685 PMC8472446

